# GP-led walk-in centre in the UK: another way for urgent healthcare provision

**DOI:** 10.1186/cc12197

**Published:** 2013-03-19

**Authors:** M Arain, J Nicholl, M Campbell

**Affiliations:** 1 The University of Sheffield, UK

## Introduction

It is evident that accident and emergency departments are overloaded with patients, which results in delays in healthcare provision [1]. A large proportion of patients consist of patients with minor illness that can be seen by a healthcare provider in a primary care setting. The aim of the study was to determine the characteristics of patients using GP walk-in services, patients' satisfaction and the effect on emergency department (ED) services.

## Methods

The survey was conducted in Sheffield and Rotherham walk-in centres over 3 weeks during September and October 2011. A self-reported, validated questionnaire was used to conduct survey on the patients presenting at these centres. We estimated that a sample size of around 400 patients from each centre was required to achieve statistically robust results. A post-visit, short questionnaire was also sent to those who agreed for the second questionnaire and provided contact details. ED data were also obtained from April 2008 to March 2010, 1 year before and 1 year after the opening of the GP walk-in centre. Data were entered and analysed in PASW Statistics 18. Ethical approval of the study was obtained from the NHS ethical review committee.

## Results

A total of 1,030 patients participated in the survey (Rotherham 501; Sheffield 529). The mean age of the participants was 32.1 years at Sheffield and 30.88 years at Rotherham. A higher proportion of users were female, around 59% at both centres. Most of the patients rated high for convenience of the centre opening hours and location (above 85%, apart from the location of Sheffield centre, which was rated high by around 72% of the research participants). Overall 93% patients were satisfied with the service at Rotherham centre and around 86% at the Sheffield centre. Based on the estimation of the monthly counts of patients attending ED and the GP walk-in centre, around 14% monthly reduction in minor attendances at ED was expected. However, ED routine data did not show any significant reduction in minor attendances as a result of the opening of the GP walk-in centre.

## Conclusion

These walk-in centres have been shown to increase accessibility to healthcare service through longer opening hours and walk-in facility. Although the effect on the reduction of patients' load at the ED is not visible as these centres cover a fraction of the population, the centre has a potential to divert patients from the ED.
